# Study on Key Aroma Compounds and Its Precursors of Peanut Oil Prepared with Normal- and High-Oleic Peanuts

**DOI:** 10.3390/foods10123036

**Published:** 2021-12-07

**Authors:** Hui Hu, Aimin Shi, Hongzhi Liu, Li Liu, Marie Laure Fauconnier, Qiang Wang

**Affiliations:** 1 Institute of Food Science and Technology, Chinese Academy of Agricultural Sciences/Key Laboratory of Agro-Products Processing, Ministry of Agriculture, Beijing 100193, China; huhui@caas.cn (H.H.); sam_0912@163.com (A.S.); lhz0416@126.com (H.L.); liulicaas@126.com (L.L.); 2 Laboratory of Chemistry of Natural Molecules, Gembloux Agro-Bio Tech, Liege University, Passage des Déportés 2, 5030 Gembloux, Belgium

**Keywords:** peanut, high-oleic, peanut oil, volatiles, precursors

## Abstract

High-oleic acid peanut oil has developed rapidly in China in recent years due to its high oxidative stability and nutritional properties. However, consumer feedback showed that the aroma of high-oleic peanut oil was not as good as the oil obtained from normal-oleic peanut variety. The aim of this study was to investigate the key volatile compounds and precursors of peanut oil prepared with normal- and high-oleic peanuts. The peanut raw materials and oil processing samples used in the present study were collected from a company in China. Sensory evaluation results indicated that normal-oleic peanut oil showed stronger characteristic flavor than high-oleic peanut oil. The compounds methylpyrazine, 2,5-dimethylpyrazine, 2-ethyl-5-methylpyrazine and benzaldehyde were considered as key volatiles which contribute to dark roast, roast peanutty and sweet aroma of peanut oil. The initial concentration of volatile precursors (arginine, tyrosine, lysine and glucose) in normal-oleic peanut was higher than in high-oleic peanut, which led to more characteristic volatiles forming during process and provided a stronger oil aroma of. The present research will provide data support for raw material screening and sensory quality improvement during high-oleic acid peanut oil industrial production.

## 1. Introduction

Peanut is one of the most important oil crops in the world. Worldwide, the production of peanuts reached 49.62 million tons in 2020/21, and the production of peanut oil was 6.43 million tons, among which approximately 50% was produced in China [1]. The total amount of unsaturated fatty acid is over 85% in peanut oil. The fatty acid profile of peanut oil resembles that of olive oil, which could reduce the risk of cardiovascular disease [2].

The flavor, nutritional quality, and shelf-life of peanut and its products are related to the relative proportion of various fatty acids [3]. With more than 72% oleic acids, high-oleic peanut is well recognized by processors for its low oxidative and ability to extend the shelf life of products [4]. Wang Qiang research group reported that high-oleic peanut oil could attenuate diet-induced Metabolic Syndrome, associated with modulating gut microbiota [5]. The breeding of high-oleic acid peanut in China has developed rapidly in recent years. Since the first high-oleic natural mutant discovered in 1987, over 190 high oleic peanut cultivars have been developed in China [6]. More and more peanut processing companies are trying to use high-oleic acid peanut in oil processing. More than five brands of high-oleic peanut oil have entered the market in China in the last three years. All these products use high-oleic runner peanut raw materials from the USA. However, the consumer feedback showed that the aroma of high-oleic peanut oil was not as good as that of normal-oleic peanut oil.

Compared with other edible vegetable oils, aromatic roasted peanut oil obtained by thermal processing is more popular for consumers because of its strong nutty and roasty flavor [7]. The unique flavors of thermally processed foods are commonly generated through the Strecker degradation during the Maillard reaction, which is responsible for generating various heterocyclic compounds, including pyrazines, pyrroles, pyridines, etc. [8]. Correlation of volatile compounds to peanut sensory evaluation has attracted researcher’s attention. A previous study reported that aspartic acid, glutamic acid, glutamine, asparagine, histidine, and phenylalanine contributed to the characteristic peanut flavor formation, and monosaccharides are highly related to pyrazine component [9]. Pyrazine compounds are responsible for the roasted flavor and aroma during peanut roasting [10]. Over 100 volatile components were identified in hot-pressed peanut oil, including pyrazines, aldehydes, furans, alcohols and pyrroles. Pyrazines are considered to be the major volatile compounds responsible for the typical roasted/nutty flavor of hot-pressed peanut oil [11]. The compounds 2/3-methyl-1H-pyrrole, 5-methyl-2-furancarboxaldehyde, benzeneacetaldehyde, 2,3 dimethyl-1H-pyrrole, 2,5 dimethyl pyrazine, 5-methyl-2-furanmethanol, and maltol were considered the most important volatile components which positively correlated with the peanutty and roasted aroma [12].

The major precursors for volatiles in peanut are proteins, sugars, and lipids [13]. Different kinds of sugars and proteins mixtures react differently, which lead to different volatiles formation. Compared with glycine and diglycine, triglycine has the highest capability to formed pyrazines in Maillard model systems. Major pyrazines were identified as 2,5-dimethylpyrazine and trimethylpyrazine [14]. Glutamine and asparagine have shown high reactivities to produce high content of pyrazines [15]. The rapeseed peptides subsequently reacted with D-xylose to largely produce methylpyrazine and ethyl-2,5-dimethylpyrazine [16]. Methylpyrazine and 2,5-dimethylpyrazine were identified in the D-glucose and L-theanine Maillard model systems but were not detectable in thermal reactions with single D-glucose or L-theanine [17]. The compounds 2,6-dimethyl-3-ethyl pyrazine, 2,5-diethylpyrazine and 2-methyl-3,5-diethylpyrazine were formed in the reaction between 1,4-13C-labeled L-ascorbic acid and L-glutamic acid. The α-amino carbonyl or α-amino hydroxy compounds were found to be the precursors of pyrazines [18].

The sensory quality difference between normal- and high-oleic peanut has also been studied. There were small differences in the roasting, astringency, over-roasting, and nuttiness attributes between these two kinds of peanuts. High-oleic lines exhibiting slightly greater intensities of those attributes [19]. Variation among individual lines for several sensory attributes (dark roasted, raw/beany, roasted peanutty, sweet aromatic, sweet, bitter, wood-hulls-skins, and “off flavors” stale/cardboard, fruity/fermented and plastic/chemical) suggest the flavor of high-oleic cultivars is at least as good as the profiles of normal-oleic cultivars [20].

Studies of characteristic volatile compounds and precursors of normal- and high-oleic peanut oil are still lacking. The object of this study was to compare the sensory quality and the key aroma components of normal- and high-oleic peanut oil produced industrially. For a possible precursor study, the amino acids and reducing sugar profile of peanut have also been monitored during oil processing. The results of this study will provide data support for raw material screening and sensory quality improvement during high-oleic acid peanut oil industrial production.

## 2. Materials and Methods

### 2.1. Materials

Normal- and high-oleic peanut raw materials and oil processing samples (roasted peanut, peanut oil, and peanut meal) were collected from the industrialized production line in factory (Jinsheng Cereals & Oils Group, Shandong Province, China). The varieties of normal- and high-oleic peanut are Baisha 1016 (China) and high-oleic runner (USA), respectively. The peanut raw material was roasted at 150 °C for 45 min. After this, roasted peanuts were pressed at 120 °C to obtain peanut oil. All reagents used in this research were obtained from Sigma-Aldrich (St. Louis, MO, USA), including methyl-pyrazine, 2,5-dimethylpyrazine, 2-ethyl-5-methylpyrazine, 1-methyl-1H-pyrrole, furfural, benzaldehyde, 2-furanmethanol, hexanal, pentanal, 1,2,3-trichloropropane, etc.

### 2.2. Sensory Evaluation

Sensory evaluation was performed at room temperature. Twelve panelists (7:5 male: female) participated to sensory evaluation. All of the panelists are well-trained researchers with a minimum of 300 h experience in sensory evaluation. Details on the methods, lexicon and attribute definitions have been previously published [21,22,23]. The sensory attributes used were roast peanutty aroma, dark roast aroma, sweet aroma, raw/beany aroma, woody/hulls/skins aroma, and a 9-point scale was used (1 = very weak, 9 = very strong). The lexicon of flavor sensory attributes is shown in Table 1.

### 2.3. Volatile Compounds Analysis

Volatile compounds in normal- and high-oleic peanut and oil processing samples were analyzed by headspace-solid phase micro-extraction (HS-SPME). SPME fiber (50/30 μm divinylbenzene/Carboxen/polydimethylsiloxane, Stableflex, Supelco Co., Bellefonte, PA, USA) was utilized for flavor extraction. The fiber was previously conditioned at 270 °C for 30 min before the first measurement. The sample (5 g) were weighed into a 20 mL glass vial which was sealed with an aluminum cover and a Teflon septum. A 25 μL aliquot of 1,2,3-trichloropropane (0.25 mg/mL in methanol) as internal standard was added. It was pre-equilibrated for 10 min at 55 °C in shaken incubator. After the equilibration time, an auto SPME holder containing fiber was inserted into the vial, and the fiber was exposed to the headspace for 40 min. The volatiles absorbed by the fiber were thermally desorbed in the hot injection port of the GC for 150 s at 260 °C. GC-MS analysis was performed using GC system (Agilent 7890B, Agilent Technologies, Santa Clara, CA, USA) and mass selective detector (Agilent 5977B) equipped with a VF-WAX column (30 m × 0.25 mm i.d., 0.25 μm film thickness; Agilent CP9205, Agilent Technologies, Santa Clara, CA, USA). The analysis was carried out in the splitless mode, using helium as the carrier gas (1 mL/min flow rate). The detector temperature was 250 °C. The oven temperature program was initially set at 40 °C for 5 min, and programmed at 5 °C/min to 250 °C which was held for 5 min. Mass spectra were recorded in electron impact ionization mode (70 eV) scanning a mass range (*m/z*) from 35 to 500 amu. The ion source temperature was maintained at 230 °C. For the identification of volatiles, the peanut oils were analyzed by GC-MS under the experimental conditions mentioned above. Volatiles were primarily identified by comparison of the mass spectra with data from the commercially available mass spectra NIST databases. In addition, the volatiles were identified by matching the retention indices (RI) with data found in the literature [24] and comparing them with commercial standards. Based on the series of n-alkanes (C7-C30), RI were calculated according to the following formula:

RIx = 100n + 100 (tRx − tRn)/(tRn + 1 − tRn)(1)

where retention time (tR) of tRn < tRx < tRn + 1; n = number of atom carbon.

### 2.4. GC-MS-O Analysis of Volatile Compounds

GC-MS-O analysis was performed using GC system (Agilent 7890B, Agilent Technologies, Santa Clara, CA, USA) and mass selective detector (Agilent 5973B) equipped with Olfactory detection port (ODP3, Gerstel, Germany). The GC-MS system parameters were the same as in 2.3. The connector temperature of the olfactometer was 150 °C. The end effluent of capillary, respectively, flows into the MS and olfactometer at a split ratio of 1:1. The odor strength was set up to a 5-point scale (1 = very weak, 5 = very strong).

### 2.5. Amino Acid Profile Analysis

Amino acids determination followed the method described in Reference [25]. The amino acid profile was measured using ion exchange chromatography. The sample (100 mg) was hydrolyzed with 10 mL 6 N HCl containing 0.1% phenol, followed by nitrogen flushing for 1 min and closing the hydrolysis bottle. Bottles were heated at 110 °C for 24 h in an oven and cooled with ice. After this, 30 mL of citrate buffer at pH 2.2 was poured (with continuous stirring) into bottles while they were still on ice. Then pH was adjusted between 0.5 and 1 using 7.5 N NaOH and then readjusted to 2.2 using 1 N NaOH. This solution was added in a 100 mL volumetric flask already containing 1 mL solution of 500 μM norleucine in citrate buffer at 2.2 pH. The volume of this flask was made 100 mL by adding citrate buffer at 2.2 pH. This solution was stirred and filtered through a 0.2 μm filter. The filtered solution was used to measure amino acids separately using Biochrom 20 plus amino acid analyzer (Biochrom Limited, Cambridge, UK).

### 2.6. Soluble Reducing Sugar Profile Analysis

Soluble reducing sugars determination followed the method described in Reference [26]. Defatted sample (0.5 g) was weighted in centrifuge tube. Soluble reducing sugars were extracted with 10 mL 70% ethanol under ultrasonic condition for 20 min. The supernatant was collected after 2000 r/min centrifugation for 10 min. The ethanol extraction and centrifuge procedure were repeated with the residue. Two parts of supernatant were filtered and vacuum rotary evaporated under 50 °C. The volume was made constant at 1 mL with 70% ethanol for analysis. The detection was performed on HPLC (Agilent 1260 Infinity, Agilent Technologies, Santa Clara, CA, USA) with diode array detector (G4212B). Spherisorb column (4.6 mm × 250 mm, 5 μm, Waters, Milford, MA, USA) was used. The mobile phase was 70% acetonitrile at a flow rate of 1 mL/min. The results were expressed as gram sugar per kilogram samples.

### 2.7. Statistical Analysis

The experiments were performed in triplicate. The least significant difference (LSD) method was used to determine the significant difference between mean values. A confidence level was set at *p* < 0.05, and the software SPSS (IBM SPSS 22.0, Chicago, IL, USA) was used for statistical analysis.

## 3. Results and Discussion

### 3.1. Sensory Evaluation of Oil Processing Samples

**Figure 1:**
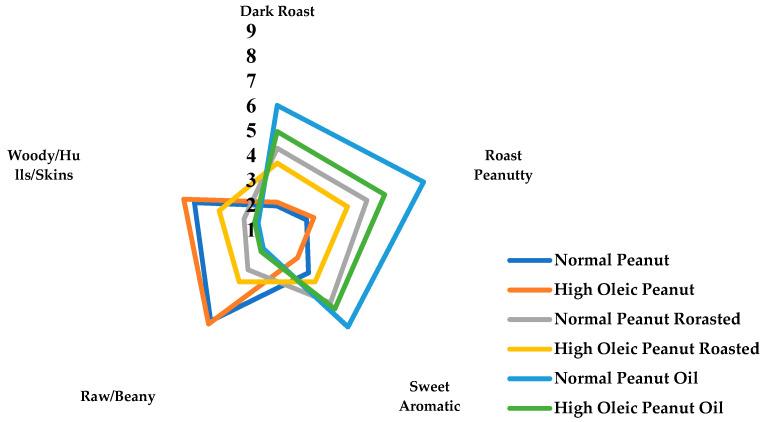
Sensory evaluation result of normal- and high-oleic peanut and oil processing samples.

Flavor is the most important quality of peanut products. The sensory evaluation results of peanut raw materials and thermal processed samples are shown in Figure 1. There are significant differences in sensory attributes between the raw material and thermal processed sample. Raw/beany and woody are the main flavors of peanut raw material. Normal-oleic peanut has a slightly stronger sweet aromatic (3.15) than high oleic peanut (2.40). Flavor attributes of high-oleic raw peanuts have been reported to be very similar to the normal oleic cultivars [19]. After roasting, raw/beany and woody flavor attributes significantly reduced. Dark roast, roast peanutty and sweet aromas make a great contribution to the roasted peanut flavor. Under the same processing conditions, roasted normal-oleic peanut has stronger roast (4.28), peanutty (4.80) and sweet (4.65) flavors, which were 16.33%, 20.75% and 29.17% higher than those of roasted high-oleic peanut, respectively. Roasted high-oleic peanuts have a stronger raw/beany (3.6) and woody (3.45) aroma than roasted normal-oleic peanuts (3 for raw/beany, 2.4 for woody). After high temperature press, the dark roast, roast peanutty and sweet aroma of samples continuously increased. Normal-oleic peanut oil has stronger roast (6.00), peanutty (7.2) and sweet (5.85) flavors, which were 21.21%, 29.73% and 18.18% higher than those of roasted high oleic peanut, respectively. The raw/beany and woody aromas of normal- and high oleic peanut oil were all around 2 with no difference. Statistically significant variation among 59 roasted peanuts was reported [20]. High oleic peanut cultivars showed a wide range of several sensory attributes (dark roasted, raw/beany, roasted peanutty, sweet aromatic, wood-hulls-skins, and “off flavors” stale/cardboard). The upper limit of positive sensory attributes for the high-oleic peanuts was greater than the normal cultivars. The differences in sensory quality between normal- and high oleic peanut products maybe caused by the composition and relative concentration of characteristic key volatile components.

### 3.2. Comparison between Volatile Components of Normal- and High Oleic Peanut Oils

As shown in Table 2, a total of 93 volatile components were identified in normal- and high-oleic peanut oil (NPO and HOPO), including 20 aldehydes, 17 alcohols, 10 alkanes, 8 acids, 5 ketones, 5 alkenes, 2 esters, 7 Pyrazines, 3 Pyridines, 3 Pyrroles, 11 furans and 2 pyrans. Most of the identified volatile components have been reported [11,24]. Several pyrazines, pyridines, pyrroles, furans and pyrans were firstly reported in the present study, including 2-methoxy-3-(1-methylethyl)-pyrazine, 1-(2-pyridinyl)-ethanone, 1-methyl-1H-pyrrole, 2-methyl-furan, 5-methyldihydro-2(3H)-furanone, 4-methyldihydro-2(3H)-furanone, 5-pentyldihydro-2(3H)-furanone, 3-hydroxydihydro-4,4-dimethyl-2(3H)-furanone, tetrahydro-2H-pyran-2-one and 3-hydroxy-2-methyl-4H-pyran-4-one. The composition and relative content of N-heterocyclic, O-heterocyclic and nonheterocyclic between normal- and high-oleic peanut oil were significantly different. The HOPO contains 39.40% N-heterocyclic, which is twice that of NPO. Among them, 35.14% 1-methyl-1H-Pyrrole in HOPO is three times that of NPO. Pyrroles were formed in the Maillard reaction and highly correlated to roast flavor and aroma [27]. Pyrazines are diverse heterocyclic nitrogen-containing compounds derived from nonenzymatic protein–sugar interactions. These volatile compounds contribute to the roasted/nutty flavor [13]. The pyrazine content of NPO and HOPO were 3.16% and 4.17%, respectively. O-heterocyclic accounting for 7.44% and 4.24% volatile components of NPO and HOPO, respectively. Ten furans in HOPO account for 6.78% of volatile components, which is twice the amount in HOPO. Furan derivatives have been identified as the second largest volatiles in roasted peanut oil [28]. They were considered to contribute to the thermally processed food flavor, including caramel-like, sweet, fruity, and nutty. Nonheterocyclic compounds were derived from lipid decomposition [29]. Aldehyde compounds were the most important nonheterocyclic compounds which appear as green, painty, metallic, beany and rancid and are also responsible for the undesirable flavors of oils [30]. The NPO contains 39.36% aldehydes which is 1.61 times that of HOPO. Hexanal accounts for 17.29% and 5.74% of total volatiles in NPO and HOPO, respectively. High oleic acid improves the oxidative stability of peanut products and reduces the formation of aldehydes.

### 3.3. Comparison between Key Volatile Components of Normal- and High Oleic Peanut Oils

The contribution of volatile components to the whole flavor of peanut oil was based on their relative concentration, odor classification and odor strength. Quantitative determination and odor strength evaluation are used for characteristic volatile components study. The correlation between characteristics volatile compounds and sensory characteristics has been studied. The compounds 2,5-dimethylpyrazine (correlated with nutty and roasted odors) and 1-methyl-1H-pyrrol (correlated with sweet and woody odor) are two of the most reported volatile components in roasted peanut products. The compounds 2/3-methyl-1H-pyrrole, 5-methyl-2-furancarboxaldehyde, benzeneacetaldehyde, 2,3-dimethyl-1H-pyrrole, 2,5-dimethylpyrazine, 5-methyl-2-furanmethanol and maltol are positively correlated to peanutty and roast aroma [31]. Having the second highest relative concentration of furan derivatives, 2-furaldehyde contributes to the sweet and caramel-like aromas of heated foods [32]. As a Strecker degradation product of phenylalanine amino acid, benzaldehyde provided an almond-like aroma [33].

As shown in Table 3, several pyrazines, pyrroles, furans and aldehydes were screened out as possible key volatiles, which contribute to the nutty, roasty and sweet flavors of peanut oil. Among all pyrazines, 2,5-dimethylpyrazine was most highly correlated to roasted peanut flavor and aroma. Comparing with 1-methy-1H-pyrrole, 3 pyrazine showed stronger nutty odor with GC-MS-O evaluation in the present study. 2,5-dimethylpyrazine has the strongest nutty odor (3.67). The nutty odor strength of methylpyrazine and 2-ethyl-5-methylpyrazine are 3.00 and 2.67. Although the 1-methyl-1H-pyrrole has the highest relative concentration (6.29 mg/kg in NPO, 7.28 mg/kg in HOPO), its nutty odor strength is only 1.33. A similar result was reported in which the correlation coefficient of 1-methyl-1H-pyrrole to nutty flavor was relatively low [31]. It can be determined that methylpyrazine, 2,5-dimethylpyrazine and 2-ethyl-5-methylpyrazine are key volatiles that contribute to the nutty and roast flavor of peanut oil. The NPO contains 0.28 ± 0.02 mg/kg methylpyrazine, 0.62 ± 0.05 mg/kg 2,5-dimethylpyrazine and 0.37 ± 0.03 mg/kg 2-ethyl-5-methylpyrazine, which are 75%, 72% and 48% higher than those of HOPO, respectively. The sensory comparison between normal- and high-oleic peanut has been reported. Compared with normal-oleic peanut, four high-oleic breeding lines (derived by the Florida high-oleic gene) showed a stronger roasted peanut sensory attribute [34]. High-oleic peanuts contribute higher roast (1.83 vs. 1.57, *p* < 0.05) and nutty (2.69 vs. 2.53, *p* < 0.05) aromas than normal-oleic peanuts among 14 peanut genetic entries (5 high-oleic, 9 normal-oleic). Roasted high-oleic peanuts have a wider roasted peanutty (3.92–5.15) odor range than roasted normal-oleic peanut (4.26–4.89) [19].

The compounds 1-methyl-1H-pyrrole, furfural, benzaldehyde, 2-furanmethanol and 3-hydroxyl-2-methyl-4H-pyran-one contribute to the sweet aroma of peanut oil (Table 3). Among of them, benzaldehyde and 3-hydroxyl-2-methyl-4H-pyran-one are considered to be the key volatiles with 2.33 and 2.00 sweet aroma strength, respectively. Compared with normal-oleic peanut oil, high-oleic peanut oil has a higher relative concentration of furfural (0.62 vs. 0.00), benzaldehyde (1.30 vs. 0.57), 2-furanmethanol (0.44 vs. 0.05) and 3-hydroxyl-2-methyl-4H-pyran-one (0.32 vs. 0.18), which lead to a stronger sweet aroma. This is consistent with the previous sensory evaluation result. There is no significant difference on sweet aroma between high-oleic roasted (2.44) and normal-oleic roasted peanuts (2.39) [19]. Sweet aromatic strength ranged from 2.41 to 3.24 fiu for high-oleics peanuts and 2.71 to 3.24 fiu for normal-oleic [20]. Hexanal is one of the primary oxidation products of linoleic acid, which contributes the green and grassy flavors to the oil. The relative content of hexanal in normal-oleic peanut oil was much higher (8.40 vs. 1.19) than in high-oleic peanut oil. This is attributed to the oxidative stability of high-oleic oil. However, the green odor strength of hexanal is very weak (1.00).

The variety and origin of collected peanut greatly influenced the sensory comparison results between normal- and high-oleic peanut samples. In the present study, with the same processing condition, normal-oleic peanut oil has a higher relative concentration of key volatile components, which contribute to stronger roasted, nutty and sweet aromas. The differences in peanut oil flavor maybe caused by the composition of volatile precursors in raw material.

### 3.4. Comparison between Amino Acids and Reducing Sugars Profile of Normal- and High Oleic Peanut Oil Processing Samples

Proteins and sugars are considered the major precursors for volatiles in peanuts. Reactivities of amino acids in Maillard model systems have drawn much attention. Dimethylpyrazine and 3-ethyl-2,5-dimethylpyrazine were largely synthesized in an aspartic acid–ascorbic acid model system [35]. Similarly, nine pyrazines were identified in the L-glutamic acid and 1,4-13C-labeled-ascorbic acid Maillard model system, and the total content of pyrazines was 63.52 mg/mol. 2,5-dimethylpyrazine (34.42 mg/mol) and ethyl-5-methylpyrazine (21.17 mg/mol) were the major pyrazines formed in the model system [18]. The structure of the N-terminal amino acid determined the overall formation of pyrazines, and the C-terminal amino acid showed less influence. The production of 2,5(6)-dimethylpyrazine and trimethylpyrazine was very high in the case of glycine, alanine or serine, whereas it was low for proline, valine or leucine [36].

The Maillard reaction between characteristic amino acids and sugars has also been studied. A quantity of 17,280 μg pyrazines was formed in a leucine (0.5 mol/L)-rhamnose (2.0 mol/L) model system, and 2-isoamyl-6-methylpyrazine (780 μg) was highly branched [37]. Eight pyrazines (0.805 mg/g of ribose) were synthesized in cysteine-ribose Maillard model system. 5H-5-methyl-6,7-dihydrocyclopentapyrazine (0.042 mg/g of ribose) was identified as a distinctive volatile component among all the pyrazines [38]. Volatile compounds formed by the reaction of 2-deoxyglucose with glutamine, glutamic acid, asparagine and aspartic acid were studied [39]. Compared with other amino acids-involved model systems, 2-deoxyglucose and asparagine generated the highest content of methylpyrazine. Results also indicated the importance of the 2-hydroxy group on glucose molecules for the effective generation of flavor compounds. A reactive Maillard reaction intermediate derived from xylose and phenylalanine was synthesized by using a stepwise increase of heating temperature. The Maillard Reaction intermediate reacted with cysteine to form various pyrazines [40].

The amino acids and reducing sugars profile of peanut samples during the oil processing were investigated in the present study. As shown in Table 4, there is no significant difference in amino acids between high-oleic peanuts and normal-oleic peanuts. Arginine, tyrosine and lysine were continuously decreased during the thermal processing. Among of them, arginine has the highest relative concentration in peanut raw materials. During the roasting procedure, the relative concentration of arginine in normal-oleic peanut decreased from 2.63 g/100 g to 1.13 g/100 g, which is also the highest loss of all the amino acids. The relative concentration of arginine in high-oleic peanuts decreased from 2.51 g/100 g to 1.08 g/100 g. There was no tyrosine detected in roasted peanut and oil samples, which indicates that all the tyrosine was reacted in the roasting procedure. As shown in Table 5, glucose was the only sugar which was continuously consumed during the thermal processing. The relative concentration of glucose in normal-oleic peanuts decreased from 0.18 mg/g to 0.12 mg/g during the thermal procedure. The content of glucose in high-oleic peanuts decreased from 0.07 mg/g to 0.03 mg/g during peanut oil processing. The relative concentration of arginine, tyrosine, lysine and glucose in peanut samples had a significant negative correlation with characteristic pyrazines, which indicated these compositions could be precursors of key volatile components. The initial relative concentration and process consumption of characteristic precursors (arginine, tyrosine, lysine and glucose) in normal-oleic peanuts was higher than in high-oleic peanuts, which led to the formation of more specific volatile components. This is consistent with sensory evaluation results for normal- and high-oleic peanut oil. Similarly, a quantity of 2229.66 mg/mol pyrazines were formed with the Maillard model system between 0.5 mol/L tyrosine and 0.5 mol/L glucose under 130 °C for 2.5 h. 2,5-Dimethylpyrazine and 2-ethyl-3-methylpyrazine were the majority of 15 formed pyrazines [35]. The effects of high-intensity ultrasound on Maillard reaction in a model system of D-xylose and L-lysine were studied [41]. 2-Methylpyrazine, 2,5-Dimethylpyrazine, 2,3-Dimethylpyrazine and 2,3,5-Trimethylpyrazine were formed in the thermal model. The ultrasonic-assisted Maillard model system could produce 3-ethyl-2,5-dimethylpyrazine, butyl amine and maltol, which were absent from thermal model. The capacity of glucose for pyrazine formation during the Maillard reaction was reported [42]. The glucose produced by Maillard reaction generated 56.7 ng/g 2-methylpyrazine, which is 18.62–32.17% higher than the fructose, ribose and xylose produced by Maillard reaction.

## 4. Conclusions

Significant differences in sensory attributes were found between peanut raw materials and thermal processed samples. Sensory evaluation results showed that normal-oleic peanut oil has a stronger dark roast, roast peanutty and sweet aroma than high-oleic peanut oil under the same processing conditions. Methylpyrazine, 2,5-dimethylpyrazine and 2-ethyl-5-methylpyrazine are considered to be the key volatiles contributing to the nutty and roasty flavor of peanut oil. Benzaldehyde and 3-hydroxyl-2-methyl-4H-pyran-one play important roles in the sweet aroma of peanut oil. The initial concentration of characteristic precursors (arginine, tyrosine, lysine and glucose) in normal-oleic peanuts was higher than in high-oleic peanuts, which led to the formation of more specific volatile components and contributed to the stronger, specific aroma of the oil. The formation mechanism of key volatiles in peanut oil needs to be further investigated. The results of this study could provide data to support the screening of suitable high-oleic peanut varieties for industrial oil processing and improve the characteristic flavor of peanut oil.

## Figures and Tables

**Table 1:** Flavor sensory attributes as obtained from the expert panel.

Sensory Attribute	Description ^a^
Roast Peanutty	The aromatic associated with medium-roast peanuts having a fragrant character such as methyl pyrazine
Dark Roast	The aromatic associated with dark roasted peanuts having a very browned or toasted character
Sweet Aromatic	The aromatics associated with sweet material such as caramel, vanilla or molasses
Raw/Beany	The aromatics associated with light roast peanuts having a legume like character
Woody/Hulls/Skins	The aromatics associated with base peanut character (absence of fragrant top notes) related to dry wood, peanut hulls and skins

^a^ Lexicon and method defined in the literature [21,22,23].

**Table 2:** Composition and relative content of volatile components in normal- and high-oleic peanut oil.

			Normal-Oleic Peanut Oil	High-Oleic Peanut Oil
Order	Retention Index	Volatile Compound	Volatile Compound (%)	Volatile Compound (%)
		**N-heterocyclic**		
		Pyrazines		
1	1265	Pyrazine, methyl-	0.57	0.79
2	1319	Pyrazine, 2,5-dimethyl-	1.27	1.73
3	1334	Pyrazine, ethyl-	0.24	0.22
4	1344	Pyrazine, 2,3-dimethyl-	0.08	0.03
5	1389	Pyrazine, 2-ethyl-5-methyl	0.75	1.20
6	1434	Pyrazine, 2-methoxy-3-(1-methylethyl)	0.14	
7	1440	Pyrazine, 3-ethyl-2,5-diemethyl	0.09	0.19
		Pyridines		
8	1178	Pyridine	0.11	
9	1577	Pyridine, 3-methoxy-	0.49	
10	1599	Ethanone, 1-(2-pyridinyl)	0.10	0.09
		Pyrroles		
11	1140	1H-Pyrrole, 1-methyl-	12.94	35.14
12	1976	Ethanone, 1-(1H-pyrrol-2-yl)	0.13	
13	2032	1H-Pyrrole-2-caboxaldehyde	0.05	
		Total	16.98	39.40
		**O-heterocyclic**		
		Furans		
14	1233	Furan, 2-methyl-	0.17	
15	1235	Furan, 2-pentyl-	2.39	1.12
16	1608	2(3H)-Furanone, dihydro-5-methyl		0.18
17	1614	2(3H)-Furanone, dihydro-4-methyl	1.04	1.05
18	1629	2(3H)-Furanone, dihydro-	0.53	0.74
19	1665	2-Furanmethanol	0.91	0.22
20	1697	Furan, 2-pentyl-	0.14	
21	1730	2,5-Furandione, 3,4-dimethyl	0.09	
22	2027	2(3H)-Furanone, dihydro-5-pentyl	0.07	
23	2042	2(3H)-Furanone, dihydro-3-hydroxy-4,4-dimethyl	0.41	
24	2407	2,3-dihydro-benzofuran	1.03	
		Pyrans		
25	1804	2H-Pyran-2-one, tetrahydro-		0.06
26	1968	4H-Pyran-4-one, 3-hydroxy-2-methyl-	0.67	0.87
		Total	7.44	4.24
		**Nonheterocyclic**		
		Aldehydes		
27	<1000	Butanal, 2-methyl-	1.19	1.12
28	<1000	Butanal, 3-methyl-	1.16	0.88
29	<1000	Pentanal	2.87	0.48
30	1078	Hexanal	17.29	5.74
31	1185	Heptanal	0.76	1.89
32	1218	2-Hexenal, (E)	0.48	0.13
33	1290	Octanal	1.08	3.75
34	1324	2-Heptenal, (Z)	4.94	1.06
35	1395	Nonanal	1.33	4.95
36	1429	2-Octenal, (E)	0.63	
37	1468	Furfural	1.27	0.76
38	1518	Benzaldehyde	2.69	2.77
39	1531	2-Nonenal, (E)	0.47	
40	1643	benzeneacetaldehyde	1.15	0.10
41	1704	Benzaldehyde, 4-ethyl-	0.12	0.09
42	1762	2,4-Decadienal	0.56	
43	1783	3-Phenylbutanal	0.13	
44	1806	2,4-Decadienal, (E,E)-	1.21	
45	1829	2-Propenal, 3-phenyl	0.05	
46	2405	Benzaldehyde, 4-methyl		0.70
		Alcohols		
47	<1000	2-Propanol		0.33
48	<1000	Ethanol	0.44	
49	<1000	2-Butanol		0.05
50	1092	1-Propanol, 2-methyl-	0.06	0.31
51	1207	1-Butanol, 3-methyl		2.16
52	1256	1-Pentanol	4.08	2.16
53	1359	1-Hexanol	5.79	2.29
54	1453	1-Octen-3-ol	2.33	0.36
55	1558	1-Octanol	0.46	2.45
56	1582	2,3-Butanediol	0.08	
57	1618	Ethanol, 2-(2-ethoxyethoxy)	0.18	0.26
58	1659	1-Nonanol		0.62
59	1791	Ethanol, 2-(2-butoxyethoxy)-	0.15	
60	1912	Phenylethyl Alcohol	1.33	2.89
61	2018	Phenol	0.08	0.07
62	2184	Phenol, 2-(1-methylpropyl)-	0.19	
63	2309	5-Thiazoleethanol, 4-methyl	0.35	
		Alkanes		
64	<1000	Pentane	6.34	
65	<1000	Heptane	1.01	1.59
66	<1000	Octane	1.92	2.00
67	<1000	Heptane, 2,4-dimethyl		0.65
68	<1000	2-Propanone		1.35
69	<1000	Octane, 4-methyl		0.43
70	<1000	Heptane, 2,2,4,6,6-pentamethyl-	0.51	4.52
71	<1000	Decane	0.03	2.29
72	1197	Dodecane		1.07
73	1401	Tetradecane	0.80	
		Acids		
74	1496	Acetic acid	1.58	0.73
75	1769	Pentanoic acid	0.35	0.11
76	1875	Hexanoic acid	1.87	0.96
77	1981	Heptanoic acid	0.44	0.49
78	2087	Octanoic acid	0.46	0.28
79	2164	Benzoic acid	0.55	
80	2192	Nonanoic acid	0.99	0.50
81	2298	Decanoic acid	0.07	
		Ketones		
82	1129	3-Penten-2-one, 4-methyl-	0.50	
83	1182	2-Heptanone	0.49	
84	1286	2-Octanone	0.14	0.20
85	1340	6-Methyl-5-hepten-2-one	0.06	0.06
86	1407	3-Octen-2-one	0.33	
		Alkenes		
87	<1000	2-Octene, (Z)	0.49	
88	<1000	2-Octene, (E)	0.23	
89	<1000	Alpha-Pinene	0.27	
90	1096	Undecane		0.42
91	1195	Limonene	0.70	0.24
		Esters		
92	1071	Acetic acid, butyl ester		0.12
93	1635	Decanoic acid, ethyl ester	0.55	
		Total	75.58	56.37

**Table 3:** Odor strength and relative concentration of key volatile components in normal- and high-oleic peanut oil.

				Normal-Oleic Peanut Oil	High-Oleic Peanut Oil
Retention Time	Key Volatile Compounds	Odor Description	Odor Strength	Concentration (mg/kg)	Concentration (mg/kg)
7.4–7.5	Pentanal	Nutty	1.33 ± 0.58	1.39 ± 0.08	0.10 ± 0.01
10.3–10.6	Hexanal	Green, Beany	1.00 ± 0.00	8.40 ± 0.74	1.19 ± 0.06
12.1–12.7	1H-Pyrrole, 1-methyl-	Nutty, Sweet	1.33 ± 0.58	6.29 ± 0.69	7.28 ± 0.42
14.9–15.1	Furan, 2-pentyl-	Green, Earthy, Beany	1.00 ± 0.00	1.16 ± 0.10	0.23 ± 0.01
16.0–16.2	Pyrazine, methyl-	Nutty, Roasted, Cocoa	3.00 ± 0.00	0.28 ± 0.02	0.16 ± 0.02
17.6–17.8	Pyrazine, 2,5-dimethyl-	Nutty, Roasted, Cocoa	3.67 ± 0.58	0.62 ± 0.05	0.36 ± 0.01
19.8–20.0	Pyrazine, 2-ethyl-5-methyl	Nutty, Roasted, Grassy	2.67 ± 0.58	0.37 ± 0.03	0.25 ± 0.00
21.3–21.5	Furfural	Sweet	1.67 ± 0.58	0.62 ± 0.05	
22.8–23.0	Benzaldehyde	Sweet	2.33 ± 0.58	1.30 ± 0.09	0.57 ± 0.01
25.8–26.0	2-Furanmethanol	Sweet	1.67 ± 0.58	0.44 ± 0.05	0.05 ± 0.00
32.4–32.6	4H-Pyran-4-one, 3-hydroxy-2-methyl-	Sweet	2.00 ± 0.00	0.32 ± 0.01	0.18 ± 0.01

**Table 4:** Amino acids profile of normal- and high-oleic peanut oil processing samples (g/100 g).

	Normal-Oleic Peanut	Roasted Normal-oleic Peanut	Normal-Oleic Peanut Meal	High Oleic Peanut	Roasted High-Oleic Peanut	High-Oleic Peanut Meal
Aspartic acid	2.83 ± 0.11 ^a^	2.80 ± 0.14 ^a^	3.37 ± 0.24 ^a^	2.65 ± 0.13 ^a^	2.75 ± 0.08 ^a^	3.10 ± 0.03 ^a^
Threonine	0.66 ± 0.07 ^a^	0.67 ± 0.04 ^a^	0.75 ± 0.04 ^a^	0.64 ± 0.05 ^a^	0.65 ± 0.07 ^a^	0.71 ± 0.05 ^a^
Serine	1.37 ± 0.06 ^a^	1.38 ± 0.07 ^a^	1.53 ± 0.03 ^a^	1.25 ± 0.09 ^a^	1.29 ± 0.12 ^a^	1.44 ± 0.09 ^a^
Glutamic acid	4.63 ± 0.12 ^ab^	4.65 ± 0.02 ^ab^	5.45 ± 0.31 ^a^	4.37 ± 0.16 ^b^	4.52 ± 0.15 ^ab^	5.08 ± 0.16 ^ab^
Proline	1.06 ± 0.05 ^a^	1.07 ± 0.06 ^a^	1.16 ± 0.14 ^a^	0.97 ± 0.04 ^a^	1.02 ± 0.04 ^a^	1.09 ± 0.05 ^a^
Glycine	1.22 ± 0.06 ^a^	1.22 ± 0.09 ^a^	1.42 ± 0.09 ^a^	1.43 ± 0.07 ^a^	1.45 ± 0.05 ^a^	1.55 ± 0.08 ^a^
Alanine	0.92 ± 0.08 ^a^	0.93 ± 0.05 ^a^	1.06 ± 0.07 ^a^	0.86 ± 0.00 ^a^	0.91 ± 0.07 ^a^	0.99 ± 0.03 ^a^
Cystine	0.35 ± 0.02 ^a^	0.35 ± 0.01 ^a^	0.39 ± 0.02 ^a^	0.35 ± 0.04 ^a^	0.35 ± 0.02 ^a^	0.40 ± 0.01 ^a^
Valine	1.05 ± 0.04 ^a^	1.04 ± 0.07 ^a^	1.23 ± 0.14 ^a^	1.05 ± 0.07 ^a^	1.09 ± 0.05 ^a^	1.21 ± 0.07 ^a^
Isoleucine	0.75 ± 0.05 ^a^	0.74 ± 0.06 ^a^	0.95 ± 0.08 ^a^	0.74 ± 0.05 ^a^	0.79 ± 0.11 ^a^	0.86 ± 0.05 ^a^
Leucine	1.55 ± 0.11 ^a^	1.55 ± 0.09 ^a^	1.87 ± 0.09 ^a^	1.57 ± 0.08 ^a^	1.56 ± 0.13 ^a^	1.72 ± 0.12 ^a^
Tyrosine	0.96 ± 0.07 ^a^	0.00 ± 0.00 ^b^	0.00 ± 0.00 ^b^	0.90 ± 0.00 ^a^	0.00 ± 0.00 ^b^	0.00 ± 0.00 ^b^
Phenylalanine	1.23 ± 0.14 ^a^	0.95 ± 0.04 ^a^	1.14 ± 0.04 ^a^	1.15 ± 0.07 ^a^	1.01 ± 0.07 ^a^	1.35 ± 0.08 ^a^
Histidine	0.71 ± 0.05 ^b^	1.25 ± 0.13 ^a^	1.43 ± 0.05 ^a^	0.70 ± 0.03 ^b^	1.25 ± 0.09 ^a^	0.80 ± 0.00 ^b^
Lysine	1.02 ± 0.07 ^a^	0.70 ± 0.03 ^b^	0.69 ± 0.03 ^b^	1.00 ± 0.04 ^a^	0.74 ± 0.03 ^b^	0.73 ± 0.04 ^b^
Arginine	2.63 ± 0.16 ^a^	1.13 ± 0.05 ^b^	1.09 ± 0.08 ^b^	2.51 ± 0.07 ^a^	1.08 ± 0.07 ^b^	1.07 ± 0.01 ^b^

Volume in a row with different superscripts were significantly different (*p* < 0.5).

**Table 5:** Sugars profile of normal- and high-oleic peanut oil processing samples (g/kg).

	Normal-Oleic Peanut	Roasted Normal-Oleic Peanut	Normal-Oleic Peanut Meal	High-Oleic Peanut	Roasted High-Oleic Peanut	High-Oleic Peanut Meal
Fructose	0.26 ± 0.06 ^b^	0.81 ± 0.09 ^a^	0.94 ± 0.05 ^a^	0.24 ± 0.10 ^b^	0.62 ± 0.04 ^ab^	0.57 ± 0.05 ^ab^
Glucose	0.18 ± 0.06 ^a^	0.14 ± 0.03 ^a^	0.12 ± 0.04 ^a^	0.07 ± 0.01 ^a^	0.04 ± 0.01 ^a^	0.03 ± 0.02 ^a^
Sucrose	50.99 ± 1.37 ^b^	58.36 ± 3.18 ^ab^	68.57 ± 0.81 ^a^	56.73 ± 3.51 ^ab^	64.15 ± 3.29 ^ab^	60.19 ± 1.27 ^ab^
Maltose	3.06 ± 0.16 ^bc^	4.31 ± 0.20 ^ab^	4.83 ± 0.34 ^a^	1.87 ± 0.23 ^d^	3.27 ± 0.34 ^bc^	2.64 ± 0.12 ^cd^
Starchyose	0.69 ± 0.06 ^b^	2.36 ± 0.37 ^ab^	3.27 ± 0.45 ^ab^	2.57 ± 0.23 ^ab^	4.53 ± 0.63 ^a^	3.05 ± 0.99 ^ab^
Raffinose	2.30 ± 0.02 ^b^	3.59 ± 0.12 ^a^	3.66 ± 0.05 ^a^	2.42 ± 0.15 ^b^	2.64 ± 0.17 ^b^	2.89 ± 0.07 ^b^

Volumes in a row with different superscripts were significantly different (*p* < 0.5).

## Data Availability

The data presented in this study are available on request from the corresponding author.
